# Diagnostic capability of different morphological parameters for primary open‐angle glaucoma in the Chinese population

**DOI:** 10.1186/s12886-021-01906-6

**Published:** 2021-03-25

**Authors:** Ruoshi Li, Xia Wang, Yahui Wei, Yuan Fang, Tian Tian, Lei Kang, Mei Li, Yu Cai, Yingzi Pan

**Affiliations:** Department of Ophthalmology, Peking University First Hospital, No.8 Xi Shi Ku Street, Xi Cheng District, 100034 Beijing, People’s Republic of China

**Keywords:** Primary open‐angle glaucoma, Novel Bruch’s membrane opening based disc parameters, Optical coherence tomography, Glaucoma diagnosis

## Abstract

**Background:**

To assess the diagnostic capability of novel Bruch’s membrane opening (BMO)-based disc parameters, the BMO-minimum rim width (BMO-MRW) and the BMO-minimum rim area (BMO-MRA) in the Chinese population and compare them to the retinal nerve fiber layer (RNFL) from optical coherence tomography (OCT) and the rim area (RA) from the Heidelberg retinal tomograph-III (HRT-III).

**Methods:**

In total, 200 eyes of 77 healthy and 123 primary open-angle glaucoma (POAG) subjects were included in this cross-sectional study. All participants underwent the visual field test and structural measurements by OCT and HRT-III. The areas under the receiver operating characteristic curves (AUCs) of different structural parameters were calculated to assess their diagnostic power and compared using the DeLong test.

**Results:**

In populations with different characteristics, the BMO-MRW and BMO-MRA had better diagnostic power than the RA. In discriminating between all POAG subjects and healthy controls and between early-stage patients and controls, the global BMO-MRW had comparable AUCs with the RNFL, but the BMO-MRA had lower AUCs than the RNFL. In healthy subjects with macrodiscs, both the global and sectoral BMO-MRW were thinner than those in healthy subjects with normal disc size. The AUCs of BMO-MRA, BMO-MRW and RNFL in subjects with macrodiscs were comparable. Additionally, in the myopic population, the BMO-MRA and BMO-MRW had comparable AUCs with the RNFL.

**Conclusions:**

The BMO-MRW had comparable diagnostic power with the RNFL, and compared with BMO-MRW, the BMO-MRA might have advantages in certain populations, such as macrodiscs. All OCT-derived parameters exceeded the RA in diagnostic capability.

## Background

Glaucoma is the leading cause of irreversible blindness worldwide, and primary open-angle glaucoma (POAG) is one of the most common types of glaucoma [1]. Detection and treatment of glaucoma at an early stage can effectively slow progression and maintain visual function. Since glaucomatous damage is characterized as optic disc damage, such as rim thinning or cup excavation and corresponding retinal nerve fiber layer (RNFL) defects, quantifying structural damage is crucial for the diagnosis and monitoring of glaucoma [2].

With the development of in vivo detection of optic discs and optical coherence tomography (OCT) techniques, several structural parameters have been introduced for the early detection of POAG [3–5]. Novel Bruch’s membrane opening (BMO)-based disc parameters were proposed recently for quantifying glaucomatous damage in the optic disc[6, 7], and are supposed to accurately reflect the rim tissue in consideration of the trajectory of nerve fibers and be stable for follow-up compared to traditional rim measurements with the application of a minimum distance algorithm and individual regionalization according to the personal BMO-fovea axis [8]. Previous studies have demonstrated that the one-dimensional parameter BMO-minimum rim width (BMO-MRW) has better diagnostic capability and a stronger correlation with visual field (VF) damage than traditional disc parameters, even surpassing the RNFL in some studies [9–13]; however, the BMO-MRW values in normal subjects were found to be related to disc size, and larger discs might have a physiologically thinner BMO-MRW, resulting in inaccurate comparability between subjects with different optic disc sizes [14, 15]. Therefore, the two-dimensional parameter BMO-MRA was introduced in 2014. Gardiner et al [7] first reported a better correlation of the BMO-MRA with the RNFL and VF defects than the rim area (RA) from the Heidelberg retinal tomograph (HRT). Other studies found that the BMO-MRA had better diagnostic power than the BMO-MRW and RNFL [7, 16, 17]. Furthermore, the BMO-MRA was demonstrated to compensate for the impact of disc size [16]. Therefore, the BMO-MRA is supposed to be more advantageous than the BMO-MRW when applied to comparison of different disc sizes; however, the BMO-MRA has thus far only been investigated in only a few studies with Caucasian populations.

In our study, we aimed to compare the diagnostic capability of the RNFL, BMO-MRW, BMO-MRA from OCT and the RA from the HRT in a Chinese POAG population and we hope that the results will provide clinicians with alternative and better parameters for the early diagnosis of POAG.

## Methods

This was a retrospective, cross-sectional study conducted in a Chinese population. POAG patients were enrolled consecutively from 02/2015 to 07/2019 at the Department of Ophthalmology, Peking University First Hospital, and healthy controls were recruited from physical examinations. This study followed the tenets of the Declaration of Helsinki and was approved by the Ethics Committee of Peking University First Hospital.Informed consent was waived since this was a retrospective observational study.

Inclusion criteria for POAG patients were: age > 18 years, best-corrected visual acuity (BCVA) 20/40 or better, refractive error within − 6 to + 5 diopters, astigmatism < 3 diopters, open angle on gonioscopy, reliable and repeatable VF test, typical glaucomatous optic damage and corresponding RNFL defects. The exclusion criteria included the followings: VF defects caused by other ocular or neural diseases, ocular trauma, intraocular surgery within 6 months, secondary glaucoma, and other diseases that might affect retinal and optic disc structures. If both eyes fulfilled the entry criteria, the less affected eye was chosen. For healthy controls, the inclusion criteria were: age > 18 years, BCVA ≥ 20/40, refractive error within − 6 to + 5 diopters, astigmatism < 3 diopters, open angle on gonioscopy, normal VF test and IOP ≤ 21mmHg. The exclusion criteria included the followings: family history of glaucoma, intraocular surgery within 6 months, and other diseases that might affect retinal and optic disc structures. One eye of each healthy subject was randomly selected. In addition, referencing a previous study [18], participants with refractive errors greater than − 2 diopters and typical myopic optic discs (with the presence of beta-type peripapillary atrophy, either sectorally or circumferentially) were defined as myopic subjects.

All participants underwent a comprehensive eye examination, including a slit lamp examination, BCVA test, refraction test, Goldmann applanation tonometry, gonioscopy, fundus photography, measurements of the central cornea thickness and axial length, a VF test and a structural assessment by the HRT (HRT III; Heidelberg Engineering, Heidelberg, Germany) and OCT (Spectralis, Heidelberg Engineering, Heidelberg, Germany).

The Swedish Interactive Threshold Algorithm (SITA) fast 24 − 2 pattern VF test (Humphrey field analyzer, Carl Zeiss Meditec Inc., Dublin, CA, USA) was performed on each subject. Glaucomatous VF damage was defined as a cluster of at least 3 nonedge points on the pattern deviation plot with a probability < 5 %, one of which was < 1 %, a Glaucoma Hemifield Test (GHT) outside normal limits, and *p* < 0.05 for the pattern standard deviation (PSD). Additionally, early-stage POAG was defined as a mean deviation (MD) of -6 dB or above. Normal VF tests were defined as GHT within normal limits and MD/PSD within 95 % confidence limits. The VF test was confirmed to be reliable at a fixation loss rate < 20 %, a false-positive rate < 15 %, and a false-negative rate < 30 %.

The total disc area and total and sectoral RA were obtained from each subject with the HRT III. An optic disc with a total disc area > 2.43 mm^2^ was defined as a macrodisc [16]. The margin of the optic disc was drawn manually and confirmed by two observers (R.L. and X.W.). The RA was calculated using a standard reference plane. An image was excluded when strong eye movement was noted or when the standard deviation value was > 50.

**Fig. 1:**
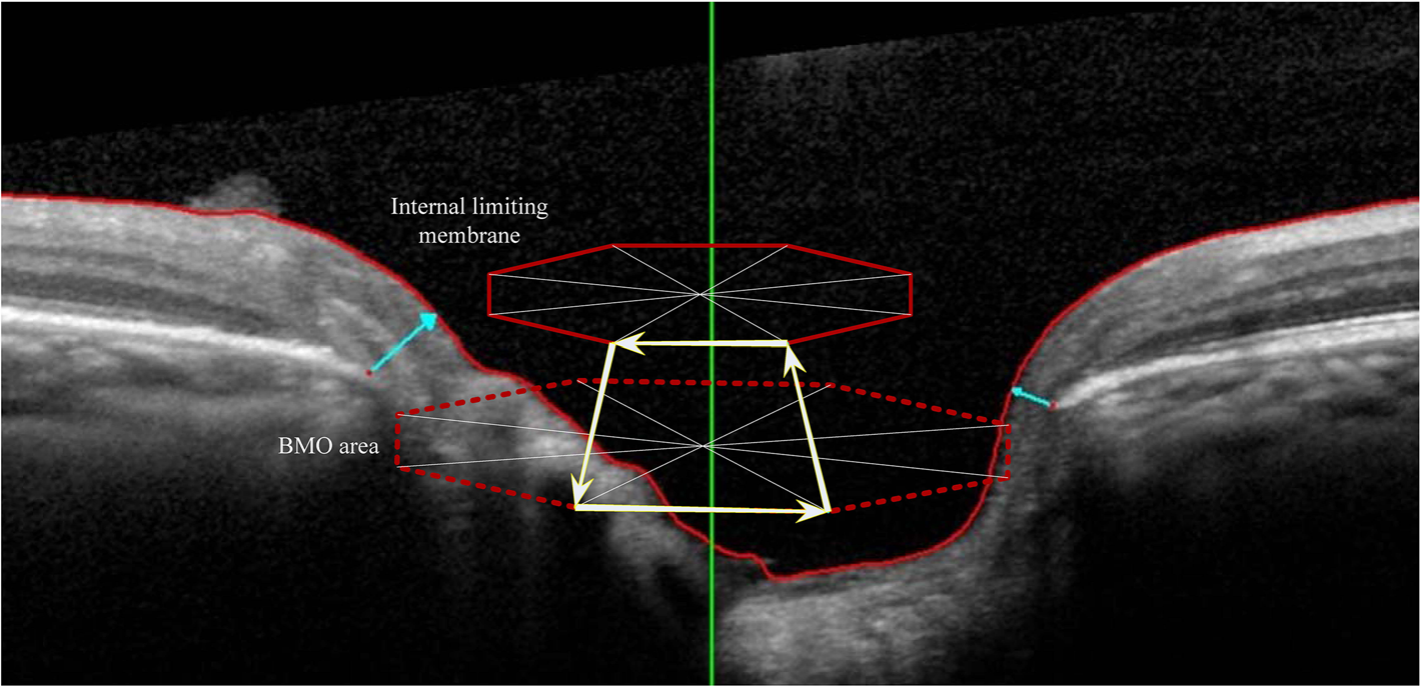
Definition and calculation of BMO-MRW and BMO-MRA. For each subject, 48 BMOs were obtained by 24 angularly equidistant radial B-scans (4 of the 24 B-scans are shown in the picture). BMO-MRW was the minimum distance between BMO and the ILM (blue arrow). BMO-MRA was the minimum surface between every two adjacent BMOs and the ILM (the area surrounded by the yellow arrows). The BMO-MRA value was determined by summing the areas of those minimum surfaces in each sector or globally

Spectralis OCT was used to measure the RNFL and BMO-based disc parameters at a wavelength of 870 nm. Before the scan, the BMO center and macular fovea were confirmed. Then, 24 angularly equidistant radial scans (BMO-MRW) and 3 annular scans (RNFL) with 3.5, 4.1, and 4.6 mm centered on the BMO center were conducted. The 3.5 mm circle was used for RNFL analysis. The internal limiting membrane (ILM) and RNFL were segmented with a segmentation algorithm and corrected manually when necessary. The BMO-MRW was defined as the minimum distance between BMO and the ILM. The BMO-MRA was calculated using Spectralis SP-X VWM (Heidelberg Engineering) freely provided by the manufacturer, and described as the minimum surface between every two adjacent BMOs and ILM, which tends to be perpendicular to the trajectories of the nerve fibers as previously described [7, 16] The BMO-MRA value was determined by summing the areas of those minimum surfaces in each sector or globally (Fig. 1). BMO was recognized automatically, then confirmed by two observers and corrected manually when wrongly marked. The BMO-MRW and BMO-MRA were divided into 6 quadrants according to the BMO-fovea axis. An image was excluded when the quality score was < 15.

### Statistical analysis

All data were analyzed using SPSS (SPSS version 20, Inc., Chicago, IL, USA) and MedCalc 15.6.1 (MedCalc software, Ostend, Belgium). All continuous variables are expressed as the mean ± standard deviation. The normality of variables was verified using Shapiro-Wilk tests. Student’s t-tests and Mann-Whitney U tests were used to compare variables between groups. The results were considered statistically significant when *p* < 0.05. Areas under the receiver operating characteristic (ROC) curves (AUCs) and sensitivities at 90 and 95 % specificity were calculated to assess the diagnostic power. The AUCs of different parameters were compared using the DeLong test [19]. Bonferroni correction was applied during multiple comparisons and the results were considered statistically significant when *p* < 0.008 (0.05/6).

## Results

In total, 200 subjects including 77 healthy controls and 123 POAG patients, were enrolled in this study, of which 73 early-stage glaucoma patients were included. All demographic and biometric characteristics are shown in Table 1. No significant differences in age, axial length, refractive error, central corneal thickness, BMO area or disc area were found between the controls and glaucoma subjects.

**Table 1:** Demographic and biometric characteristics in glaucoma and healthy group

	Controls(*n* = 77)	POAG(*n* = 123)	Early-stage POAG(*n* = 73)	p*	p†
Age (years)	55.2 ± 8.8	56.8 ± 12	54.5 ± 11.5	0.064	0.962
Gender (Male/Female)	24/53	62/61	39/34	0.008	0.008
Refractive error (diopters)	-1.02 ± 1.92	-1.34 ± 2.37	-1.36 ± 2.31	0.571	0.654
Axial length (mm)	23.81 ± 0.96	24.04 ± 1.39	24.09 ± 1.33	0.4	0.143
Central corneal thickness (µm)	541.8 ± 34	535.8 ± 34.7	537.1 ± 34.6	0.227	0.397
Mean deviation (MD, dB)	-1.39 ± 1.03	-6.81 ± 4.88	-3.67 ± 1.53	< 0.001	< 0.001
Visual Field Index (VFI, %)	98.73 ± 1.14	84.41 ± 14.53	93.1 ± 4.26	< 0.001	< 0.001
Intraocular pressure (IOP, mmHg)	14.6 ± 2.9	15.3 ± 2.8	15.4 ± 2.8	0.241	0.219
BMO area	2.36 ± 0.47	2.38 ± 0.46	2.44 ± 0.47	0.741	0.27
Total disc area	2.4 ± 0.54	2.41 ± 0.53	2.46 ± 0.5	0.876	0.379

*POAG *primary open-angle glaucoma, *MD* mean deviation, *VFI *visual field index, *IOP* intraocular pressure, *BMO* Bruch’s membrane opening

p* represent for comparison between controls and POAG, p† represent for comparison between controls and early-stage POAG

The mean disc area from the HRT was 2.4 ± 0.54 mm^2^ and 2.41 ± 0.53 mm^2^ in the healthy and glaucoma groups, respectively. The optic discs in 34 controls and 53 POAG patients were defined as macrodiscs. There were no significant differences in demographic or biometric characteristics between the healthy and glaucoma subjects with macrodiscs. In healthy subjects, the global BMO-MRW in macrodiscs (256.9 ± 33.7 μm) was significantly thinner than that in subjects with a normal disc size (288.8 ± 40.7 μm, *p* < 0.001), and statistically significant differences in the sectoral BMO-MRW were also found (*p* < 0.05); however, no significant differences in either the global or sectoral BMO-MRA were observed between subjects with different disc sizes (Table 2). Similarly, in POAG patients with different disc sizes, the global BMO-MRW in macrodiscs (182.8 ± 31.8 μm) was also significantly thinner than that in subjects with a normal disc size (197.9 ± 43.6 μm, *p* = 0.028), while no significant difference in global BMO-MRA was found.

**Table 2:** Morphological values in different groups

	Global/Total	Infero-temporal	Supero-temporal	Temporal	Infero-nasal	Supero-nasal	Nasal
**Controls(disc size < 2.43 mm**^**2**^**)**							
BMO-MRW(µm)	288.8 ± 40.7	321.9 ± 53.3	291.6 ± 48.4	199.9 ± 37.5	356.7 ± 61.3	328.7 ± 62.4	308.9 ± 53.1
BMO-MRA(mm^2^)	1.31 ± 0.23	0.17 ± 0.03	0.14 ± 0.03	0.23 ± 0.05	0.18 ± 0.37	0.16 ± 0.04	0.41 ± 0.08
RNFL(µm)	104.4 ± 11.4	162 ± 20.5	139.1 ± 20.2	79.9 ± 12.2	119.7 ± 25.5	120.3 ± 28.1	79.8 ± 15.5
Rim area(mm^2^)	1.37 ± 0.24	0.19 ± 0.05	0.17 ± 0.04	0.2 ± 0.05	0.22 ± 0.04	0.2 ± 0.05	0.38 ± 0.09
**Controls(disc size > 2.43 mm**^**2**^**)**							
BMO-MRW(µm)	256.9 ± 33.7^a^	296.9 ± 37.8^a^	258.7 ± 40.6^a^	180.6 ± 29.3^a^	322.9 ± 46.9^a^	300.6 ± 46.5^a^	262.9 ± 47.6^a^
BMO-MRA(mm^2^)	1.36 ± 0.23	0.18 ± 0.03	0.14 ± 0.03	0.24 ± 0.05	0.19 ± 0.03	0.18 ± 0.03	0.41 ± 0.08
RNFL(µm)	106.1 ± 9.3	164.8 ± 17.9	138.6 ± 21.3	75.7 ± 8.6	122.5 ± 22.1	131.5 ± 26.3	82.9 ± 10.9
Rim area(mm^2^)	1.67 ± 0.32^a^	0.21 ± 0.06	0.2 ± 0.05^a^	0.22 ± 0.06	0.28 ± 0.06^a^	0.25 ± 0.06^a^	0.48 ± 0.13^a^
**POAG**							
BMO-MRW(µm)	191.4 ± 39.5^b^	168.6 ± 64.9^b^	180.8 ± 54.9^b^	148.2 ± 33.7^b^	211.9 ± 62.8^b^	228.1 ± 61.7^b^	218.4 ± 56.9^b^
BMO-MRA(mm^2^)	0.95 ± 0.19^b^	0.1 ± 0.04^b^	0.1 ± 0.03^b^	0.18 ± 0.05^b^	0.12 ± 0.03^b^	0.12 ± 0.03^b^	0.32 ± 0.08^b^
RNFL(µm)	75.6 ± 13.9^b^	84.9 ± 36.9^b^	93.6 ± 32.1^b^	62.3 ± 12.4^b^	85.5 ± 26.9^b^	96.7 ± 29.2^b^	65.6 ± 16.4^b^
Rim area(mm^2^)	1.21 ± 0.37^b^	0.13 ± 0.07^b^	0.15 ± 0.06^b^	0.17 ± 0.08^b^	0.2 ± 0.07^b^	0.19 ± 0.06^b^	0.36 ± 0.12^b^

*BMO-MRW* Bruch;s membrane opening-minimum rim width, *BMO-MRA* Bruch’s membrane opening-minimum rim area, *RNFL* retinal nerve fiber layer, *POAG* primary open-angle glaucoma

^a^represent for statistical difference between controls with disc size < 2.43mm^2^ (macrodiscs) and controls with disc size > 2.43 mm^2 ^

^b^represent for statistical difference between controls and POAG patients

In distinguishing POAG subjects from healthy controls, the AUC and sensitivity at 90 % specificity were 0.931 and 82.9 % for the BMO-MRW, 0.901 and 75.6 % for the BMO-MRA, 0.954 and 87 % for the RNFL, and 0.74 and 43.1 % for the RA, respectively (Table 3). No significant difference was found between the AUC of the BMO-MRW and RNFL using the DeLong test (*p* = 0.176). The AUC of the BMO-MRA was comparable to that of the BMO-MRW (*p* = 0.072) but significantly smaller than that of the RNFL (*p* = 0.002). In the sectoral analysis (as shown in Table 4), the parameters in the inferotemporal quadrants had the highest AUCs among all structural parameters. The AUCs of both the BMO-MRW and BMO-MRA were comparable to that of the RNFL in most quadrants, and the BMO-MRW and BMO-MRA had higher AUCs and sensitivities at 90 and 95 % specificity than the RNFL in the inferonasal quadrants (*p* < 0.008). All OCT parameters had better diagnostic capability than the RA from the HRT both globally and regionally.

**Table 3:** Overall diagnostic power and comparison between different parameters using DeLong test

	AUC	95 % confidence intervals)	Sentivity at 90 % specificity	Sentivity at 95 % specificity
Global RNFL	0.954	(0.93,0.979)	87	79.7
Global BMO-MRW	0.931	(0.896,0.965)	82.9	69.1
Global BMO-MRA	0.901	(0.86.0.942)	75.6	61.8
Global rim area	0.74	(0.673,0.808)	43.1	35
**DeLong- test for AUCs**				
	AUC1		AUC2	p
Global BMO-MRW	0.931	Global BMO-MRA	0.901	0.072
Global BMO-MRW	0.931	Global RNFL	0.954	0.176
Global BMO-MRW	0.931	Total rim area	0.74	< 0.001
Global BMO-MRA	0.901	Global RNFL	0.954	0.002
Global BMO-MRA	0.901	Total rim area	0.74	< 0.001
Global RNFL	0.954	Total rim area	0.74	< 0.001

The results were considered statistically significant when *p* < 0.008

*AUC* area under the curve, *BMO-MRW* Bruch;s membrane opening-minimum rim width, *BMO-MRA* Bruch’s membrane opening-minimum rim area, *RNFL* retinal nerve fiber layer

**Table 4:** AUCs and sensitivities in different quadrants

	AUC(95 % confidence intervals)	Sentivity at 90 % specificity	Sentivity at 95 % specificity
BMO-MRW			
Inferotemporal	0.956(0.932,0.98)	85.4	81.3
Superotemporal	0.908(0.869,0.947)	76.4	69.9
temporal	0.814(0.757,0.872)	56.1	44.7
Inferonasal	0.937(0.905,0.969)	82.9	73.2
Superonasal	0.856(0.805,0.906)	65.9	60.2
Nasal	0.814(0.754,0.873)	58.5	53.7
BMO-MRA			
Inferotemporal	0.93(0.897,0.964)	82.1	75.6
Superotemporal	0.873(0.827,0.92)	65.9	59.3
temporal	0.754(0.688,0.82)	45.5	34.1
Inferonasal	0.918(0.882,0.955)	78	66.7
Superonasal	0.831(0.775,0.887)	50.4	42.3
Nasal	0.796(0.732,0.86)	47.2	27.6
RNFL			
Inferotemporal	0.957(0.93,0.984)	91.1	90.2
Superotemporal	0.878(0.831,0.925)	71.5	59.3
temporal	0.84(0.785,0.894)	66.7	47.2
Inferonasal	0.849(0.797,0.901)	64.2	58.5
Superonasal	0.762(0.696,0.828)	40.7	34.1
Nasal	0.77(0.704,0.836)	43.9	32.5
Rim area			
Inferotemporal	0.804(0.743,0.864)	58.5	44.7
Superotemporal	0.709(0.637,0.78)	35.8	31.7
temporal	0.683(0.61,0.756)	37.4	32.5
Inferonasal	0.701(0.628,0.773)	39	20.3
Superonasal	0.648(0.571,0.726)	26	20.3
Nasal	0.642(0.564,0.721)	28.5	17.1

*AUC* area under the curve, *BMO-MRW *Bruch;s membrane opening-minimum rim width, *BMO-MRA* Bruch’s membrane opening-minimum rim area, *RNFL* retinal nerve fiber layer

While discriminating between early-stage POAG patients and controls, all structural parameters showed lower AUCs and sensitivities than those between all glaucoma patients and controls (Table 5). Similarly, the AUC of the global BMO-MRW was comparable to that of the RNFL (*p* = 0.29), but the BMO-MRA had lower AUCs than the RNFL (*p* = 0.004). As shown in Table 6, while assessing the diagnostic capability in subjects with macrodiscs, the AUCs of the BMO-MRW and BMO-MRA increased, but no statistically significant differences were found between the AUCs of the global BMO-MRW, BMO-MRA and RNFL. All OCT parameters also surpassed the RA from the HRT in AUCs and sensitivities. Additionally, we assessed the diagnostic power in myopic subjects, including 18 healthy controls and 45 POAG patients (the demographic data were comparable between healthy and glaucoma subjects), and found that the BMO-MRW, BMO-MRA and RNFL had comparable AUCs and sensitivities, as shown in Table 7.

**Table 5:** AUCs and sensitivities to distinguish early-stage POAG from healthy subjects and comparison between different parameters using DeLong test

ROC	AUC	95 % confidence intervals)	Sentivity at 90 % specificity	Sentivity at 95 % specificity
Global RNFL	0.933	(0.897,0.969)	82.2	71.2
Global BMO-MRW	0.907	(0.859,0.954)	76.7	57.5
Global BMO-MRA	0.859	(0.801,0.917)	65.8	46.6
Global rim area	0.69	(0.605,0.775)	37	30.1
**DeLong- test for AUCs**				
	AUC1		AUC2	p
Global BMO-MRW	0.907	Global BMO-MRA	0.859	0.03
Global BMO-MRW	0.907	Global RNFL	0.933	0.29
Global BMO-MRW	0.907	Total rim area	0.69	< 0.001
Global BMO-MRA	0.859	Global RNFL	0.933	0.004
Global BMO-MRA	0.859	Total rim area	0.69	< 0.001
Global RNFL	0.933	Total rim area	0.69	< 0.001

The results were considered statistically significant when *p* < 0.008

*AUC* area under the curve, *POAG *primary open-angle glaucoma, *BMO-MRW* Bruch;s membrane opening-minimum rim width, *BMO-MRA* Bruch’s membrane opening-minimum rim area, *RNFL* retinal nerve fiber layer

**Table 6:** AUCs and sensitivities in macrodiscs and comparison between different parameters using DeLong test

ROC	AUC	95 % confidence intervals)	Sentivity at 90 % specificity	Sentivity at 95 % specificity
Global RNFL	0.951	(0.912,0.99)	87	79.7
Global BMO-MRW	0.946	(0.9,0.993)	82.9	69.1
Global BMO-MRA	0.921	(0.865,0.977)	75.6	61.8
Global rim area	0.768	(0.669,0.867)	43.1	35
**DeLong- test for AUCs**				
	AUC1		AUC2	p
Global BMO-MRW	0.946	Global BMO-MRA	0.921	0.091
Global BMO-MRW	0.946	Global RNFL	0.951	0.835
Global BMO-MRW	0.946	Total rim area	0.768	< 0.001
Global BMO-MRA	0.921	Global RNFL	0.951	0.212
Global BMO-MRA	0.921	Total rim area	0.768	< 0.001
Global RNFL	0.951	Total rim area	0.768	< 0.001

The results were considered statistically significant when *p* < 0.008

*AUC* area under the curve, *BMO-MRW* Bruch;s membrane opening-minimum rim width, *BMO-MRA* Bruch’s membrane opening-minimum rim area, *RNFL* retinal nerve fiber layer

**Table 7:** Diagnostic power in myopic subjects

ROC	AUC	95 % confidence intervals)	Sentivity at 90 % specificity	Sentivity at 95 % specificity
Global BMO-MRW	0.945	(0.895,0.995)	84.4	82.2
Global RNFL	0.943	(0.888,0.997)	77.8	77.8
Global BMO-MRA	0.934	(0.876,0.991)	77.8	77.8
Global rim area	0.698	(0.564,0.831)	53.3	33.3
**DeLong- test for AUCs**				
	AUC1		AUC2	p
Global BMO-MRW	0.945	Global BMO-MRA	0.934	0.648
Global BMO-MRW	0.945	Global RNFL	0.943	0.939
Global BMO-MRW	0.945	Total rim area	0.698	< 0.001
Global BMO-MRA	0.934	Global RNFL	0.943	0.773
Global BMO-MRA	0.934	Total rim area	0.698	< 0.001
Global RNFL	0.943	Total rim area	0.698	< 0.001

The results were considered statistically significant when *p* < 0.008

*AUC* area under the curve, *BMO-MRW* Bruch;s membrane opening-minimum rim width, *BMO-MRA* Bruch’s membrane opening-minimum rim area, *RNFL* retinal nerve fiber layer

## Discussion

The available literature on novel BMO-based disc parameters, especially the BMO-MRA, have included mostly Caucasian populations, Therefore, in this study, the diagnostic capability of both the BMO-MRW and BMO-MRA was first assessed and compared to that of conventional parameters, the RNFL from OCT and the RA from the HRT in a Chinese population.

Consistent with most previous studies [10, 12, 17, 20], we found that the global BMO-MRW and RNFL had comparable capability in distinguishing POAG subjects from healthy subjects as well as in the detection of early-stage POAG patients (Tables 3 and 5). For the BMO-MRA, we found that the global BMO-MRA offered lower diagnostic power than the RNFL (Tables 3 and 5). However, as presented in Table 4, sectoral analysis showed that both the BMO-MRA and BMO-MRW yielded a comparable diagnostic performance with the RNFL in the supero-nasal, supero-temporal and infero-temporal quadrants (*p* > 0.008) and surpassed the RNFL in the infero-nasal quadrants (*p* < 0.008). These findings differed from previous studies: Enders et al[16] found that the BMO-MRA had better diagnostic power than the BMO-MRW and RNFL in subjects with different disc sizes, and recently, the authors demonstrated that the BMO-MRA, BMO-MRW and RNFL offered a comparable level of diagnostic capability in a large cohort [17]. Since the diagnostic capability of the BMO-MRA in previous studies varied between different cohorts, we speculate that the relatively inferior diagnostic power of the global BMO-MRA in our study in comparison with previous findings might be ascribed to the different disease stages of the glaucoma patients enrolled in the different studies. In addition, combined with global and sectoral findings, the inferior diagnostic capability of the global BMO-MRA might also be related to the algorithm: the global BMO-MRA was the sum of each sector, while the global BMO-MRW and RNFL were the average of sectoral values. Therefore, we speculate that the value and diagnostic performance of the global BMO-MRA might be influenced more by the nasal and temporal quadrants than other parameters, which resulted in relatively low diagnostic power. On the other hand, similar to previous findings, we also found that both the BMO-MRW and BMO-MRA performed significantly better than the RA from the HRT in AUCs and sensitivities both globally and regionally, indicating that novel BMO-based disc parameters might be preferred options for the assessment of optic disc damage.

Additionally, we divided the subjects according to disc size and assessed diagnostic capability in macrodiscs and found that both the BMO-MRA and BMO-MRW yielded similar performances with the RNFL in differentiating POAG subjects from controls, as shown in Table 6. However, we found that both the global and sectoral BMO-MRW in healthy subjects with macrodiscs were significantly thinner than those in healthy subjects with a normal disc size, while the BMO-MRA was comparable between subjects with different disc sizes (Table 2). These findings are in accordance with Enders’s studies [16], which demonstrated that the BMO-MRA could compensate for the influence of disc size, indicating that the BMO-MRA might represent a better tool than the BMO-MRW in the management of subjects with macrodiscs and more suitable in subjects with a wide range of disc sizes.

Myopia is a widely accepted possible risk factor for POAG, but the diagnosis of POAG in myopia with available structural parameters, such as the RNFL and traditional rim parameters, remains challenging [21, 22]. Previous studies have reported that the BMO-MRW had comparable diagnostic power and a stronger correlation with VF damage than the RNFL in myopic subjects [18, 23], showing the potential capability of the BMO-MRW in distinguishing POAG in myopic subjects, but the diagnostic power of the BMO-MRA was not investigated. Therefore, referencing Malik’s study [18], as presented in Table 7, we compared the diagnostic performance of the BMO-MRW and BMO-MRA with conventional morphological parameters in myopic participants with refraction errors greater than − 2 diopters and typical myopic optic discs. Similarly, we found that both the BMO-MRW and BMO-MRA had comparable diagnostic capability with the RNFL, further indicating the possible utility of novel BMO-based disc parameters in the myopic population.

Our study has some limitations. First, the sample size in our study was relatively small, especially the number of subjects with macrodiscs and myopia, and all patients were recruited from our clinical center, which might result in selection bias. Additionally, data from the relatively few glaucoma subjects included in our study do not represent the full spectrum of the disease. Therefore, the diagnostic performance of novel BMO-based disc parameters in the Chinese population must be verified in a larger population with a full range of disc sizes from multiple centers. Second, the BMO-MRA was calculated based on the minimum surface between BMO and the ILM in each sector and therefore might not reflect the actual minimum area where neural tissue passes through. Further modifications of the calculation might be needed for evaluation in future studies.

In conclusion, in this single-center Chinese cohort study, the BMO-MRW had comparable diagnostic power with the RNFL, and the BMO-MRA could compensate for the influence of disc size and offer similar capability with the BMO-MRW and RNFL in subjects with macrodiscs and myopia. All OCT-derived parameters exceeded the RA from the HRT in diagnostic capability. Novel BMO-based morphological parameters might serve as additional tools in the clinical management of glaucoma.

## Data Availability

The datasets used and/or analysed during the current study are not publically available and can be obtained from the corresponding author on reasonable request.
